# Resistance of bone marrow mesenchymal stem cells in a stressed environment - Comparison with osteocyte cells

**DOI:** 10.7150/ijms.52104

**Published:** 2021-01-21

**Authors:** Miyako Shimasaki, Shusuke Ueda, Toru Ichiseki, Hiroaki Hirata, Norio Kawahara, Yoshimichi Ueda

**Affiliations:** 1 Department of Pathology 2, Kanazawa Medical University, Daigaku 1-1, Uchinada, Kahoku-gun, Ishikawa, 920-0293, Japan.; 2 Department of Orthopaedic Surgery, Kanazawa Medical University, Daigaku 1-1, Uchinada-machi, Kahoku-gun, Ishikawa 920-0293, Japan.

**Keywords:** mesenchymal stem cell (MSC), mitochondrial function, oxidative injury, apoptosis

## Abstract

**Introduction:** Recently, the efficacy of mesenchymal stem cells (MSCs) mediated by their tissue repair and anti-inflammatory actions in the prevention and therapy of various disorders has been reported. In this research, our attention was focused specifically on the prevention and therapy of glucocorticoid-induced osteonecrosis. We investigated the stress resistance of MSC against glucocorticoid administration and hypoxic stress, which are factors known to induce osteocytic cell death.

**Materials and Methods:** Mouse bone cells (MLO-Y4) and bone-marrow derived mouse MSCs were exposed to dexamethasone (Dex), hypoxia of 1% oxygen or both* in vitro*. Mitochondrial membrane potentials were estimated by mitochondria labeling with a cell-permeant probe (Mito tracker red); expression of these apoptosis-inducing molecules, oxidative stress marker (8-hydroxy-2'-deoxyguanosine), caspase-3, -9, and two apoptosis-inhibiting molecules, energy-producing ATP synthase (ATP5A) and X-linked inhibitor of apoptosis protein (XIAP), were analyzed by both immunofluorescence and western blot.

**Results:** With exposure to either dexamethasone or hypoxia, MLO-Y4 showed reduced mitochondrial membrane potential, ATP5A and upregulation of 8-OHdG, cleaved caspases and XIAP. Those changes were significantly enhanced by treatment with dexamethasone plus hypoxia. In MSCs, however, mitochondrial membrane potentials were preserved, while no significant changes in the pro-apoptosis or anti-apoptosis molecules analyzed were found even with exposure to both dexamethasone and hypoxia. No such effects induced by treatment with dexamethasone, hypoxia, or both were demonstrated in MSCs at all.

**Discussion:** In osteocyte cells subjected to the double stresses of glucocorticoid administration and a hypoxic environment osteocytic cell death was mediated via mitochondria. In contrast, MSC subjected to the same stressors showed preservation of mitochondrial function and reduced oxidative stress. Accordingly, even under conditions sufficiently stressful to cause the osteocytic cell death *in vivo*, it was thought that the function of MSC could be preserved, suggesting that in the case of osteonecrosis preventative and therapeutic strategies incorporating their intraosseous implantation may be promising.

## Introduction

Mesenchymal stem cells (MSCs) show a cluster action, and manifest tissue repair and anti-inflammatory activities at sites of injury, and moreover have regenerative and improvement properties that become active in the presence of injured or defective tissues. Because of their ease of administration, the clinical application of MSC in the therapy and prevention of a variety of conditions is being actively pursued. Already, reports have described the efficacy of methods using MSC in both the prevention and therapy of a wide spectrum of conditions including hepatic, pancreatic, neurogenic, cardiac, and ischemic brain diseases 1-3.

Glucocorticoid-induced osteonecrosis is a difficult condition to treat, and is associated with a markedly impaired quality of life (QOL). And despite extensive and diverse research devoted to aspects such as elucidation of its pathophysiology, therapy, and prevention, its underlying pathogenetic mechanisms remain as before unclear. Ischemic-hypoxic events are recognized to be an important triggering factor, and both oxidative injury and mitochondrial injury have been implicated in the process culminating in the necrosis of osteocyte cells 4-6. Recently, in a domestic rabbit osteonecrosis model the administration of MSC was reported to inhibit the development of osteonecrosis 7, suggesting maintenance of normal MSC function even at sites of injury exposed to ischemia-hypoxia. Indeed, the activity of MSC has been documented to be preserved or even enhanced in an hypoxic environment 8. For these reasons the functional preservation of MSC when exposed to hypoxic stress and/or oxidative stress generated by glucocorticoid administration continues to arouse major attention in the medical community.

Meanwhile, angiogenesis-osteogenesis coupling injury has been implicated in the development of osteocytic cell necrosis, with this *in vivo* environment of glucocorticoid-induced osteonecrosis reported to be reproducible *in vitro* by the addition of glucocorticoid to cultured osteocyte cells placed in a hypoxic environment 9. But before any prophylactic or therapeutic effects against glucocorticoid-induced osteonecrosis by MSC implantation can be anticipated, it is extremely important that the maintenance of MSC function in the same environment be clearly documented to be feasible.

This background prompted us to undertake the present experiment, in which the state of functional preservation of MSC in an hypoxic environment to which glucocorticoid was added, which is considered to replicate the intraosseous environment at the time of glucocorticoid administration, was investigated.

## Materials and Methods

### Cell culture, exposed conditions and counting of viable cells

An established murine osteocyte cell line (MLO-Y4) (Kerafast, Boston, MA) was maintained as a subconfluent monolayer culture in the MEM alpha medium (Gibco, Tokyo, Japan) supplemented with 10% fetal calf serum 10. Mouse MSC derived from the bone marrow (Cyagen, Silicon Valley, CA) was maintained in the stem cell growth medium (Cyagen). Both cell lines were cultured at 37°C under 20% O_2_ and 5% CO_2_. At the culture reached 80% confluency while being cultured at 37°C under 20% O_2_ and 5% CO_2_, both cell lines, MLO-Y4 and MSC, were treated under three different conditions for 24 hours: exposed to dexamethasone (Dex, MSD, Tokyo, Japan) at the concentration of 0.4 ng/ml (Dex group); hypoxia at a 1% oxygen concentration (Hypoxia group) or both (Dex/Hypoxia group). As a control (C group), both cell lines were cultured under 20% oxygen in the culture medium without dexamethasone. After the exposure, the numbers of viable and nonviable cells determined using 0.25% trypan blue dye exclusion method were counted with Countess 2 FL (Thermo Fisher Scientific, Waltham, MA), and survival rates were calculated.

### Mitochondrial membrane potential

Cultured cells were stained with Mito Tracker Red CMXRos (Thermo Fisher Scientific) at a final concentration of 200 nM in culture medium at 37°C under 20% O_2_ and 5% CO_2_ for 30 minutes 11,12. Cells were then fixed with 4% paraformaldehyde; nuclei were stained with 4', 6-diamidino-2-phenylindole (DAPI). Representative five fields were photographed using a Zeiss-LSM710 camera (Zeiss, Baden-Württemberg, Germany). Ratios of mitochondrial membrane potential positive cells were calculated by division with cell numbers stained with DAPI.

### Cell viability assay

Viability assays were then performed using an Apoptotic/Necrotic Cells Detection Kit (PromoKine, Heidelberg, Germany) according to the manufacturer's instructions, and the percentages of apoptotic/necrotic cells relative to the total cell number were determined. In the viability assays, apoptotic cells can be detected by the staining with fluorescein-labeled annexin V (green fluorescence) and necrotic cells by the staining with Ethidium homodimer III, a highly positively charged nucleic acid probe, which is impermeant to live cells and early apoptotic cells, but stains necrotic cells and late apoptotic cells (entering into secondary necrosis) with red fluorescence. Fluorescence-positive cells were evaluated by phase contrast and fluorescence (470 nm and 530 nm LED modules) microscopy using BZ-X700 (Keyence, Tokyo, Japan).

### Immunostaining for ATP synthase, 8-hydroxy-2'-deoxyguanosine, X linked inhibitor of apoptosis protein, and cleaved-caspase 3

Cultured cells were fixed in 4% paraformaldehyde, washed in phosphate buffered saline (PBS), and permeabilized with 0.3% Triton X-100 in PBS. Nonspecific binding was blocked by incubating sections with 10% bovine serum albumin (Dako Cytomation, Santa Clara, CA) in PBS for 15 minutes. They were incubated with anti-ATP synthase (ATP5A) (Proteintech, IL), anti-8-hydroxy-2'-deoxyguanosine (8-OHdG) (Abcam, Cambridge, U K), anti-X-linked inhibitor of apoptosis protein (XIAP) (Abcam), or anti-caspase-3 (Proteintech) antibody for 2 hours at a concentration of 10.0, 2.5, 10.0, or 1.5 µg/ml, followed by a fluorescent-labeled secondary antibody (for ATP5A, 8-OHdG, and XIAP, Alexa 488, Thermo Fisher Scientific; for caspase-3, Alexa 594) and by DAPI for 30 minutes. After washing, a prolong diamond antifade mountant (Thermo Fisher Scientific) was added, and cover slips were mounted. Images were taken using Zeiss-LSM710. For ATP5A, cultured cells were labeled with the first antibody after labeling with the mitochondrial membrane potential-dependent probe.

### Western blot

Protein was extracted using protein extraction solution (PRO-PREP, iNtRON Biotechnology, Kyungki-Do, Korea). The protein, 20 µg, was electrophoresed on a 10% polyacrylamide gel, and transferred to a nitrocellulose membrane (Atoh, Tokyo, Japan). The membranes were reacted overnight at 4°C with the primary antibodies. The primary antibodies applied were anti-ATP5A (Proteintech), anti-8-OHdG (ABBIOTEC, San Diego, CA), anti-XIAP (Abcam), anti-cleaved-caspase-3 (Abcam), or anti-caspase-9 (Proteintech) at a concentration of 0.5, 2.5, 1.0, 1.0, or 1.0 µg/ml. After the incubation with peroxidase-labeled goat anti-mouse or anti-rabbit IgG antibody (Dako Cytomation) for 1 hour at room temperature and vigorous washing, the nitrocellulose membrane was incubated with Chemiluminescence Luminol Reagent (Immuno Star LD, Wako, Tokyo, Japan) and photographed digitally using ImageQuant LAS 4000 mini (GE healthcare Japan Co, Tokyo, Japan). Immunoblot using anti-actin monoclonal antibody (Sigma Chemical Co. St. Louis, MO) was used for standardization. Intensity was measured using the Multi Gauge v3.1 (Fujifilm, Tokyo, Japan). Experiments were repeated at least three times.

### Statistical analysis

All data are presented as means ± SD. Differences between groups were assessed using the one-way analysis of variance followed by Fisher's protected least significant difference post hoc test. Differences were considered significant at *p* < 0.05.

## Results

### Survival rates of MLO-Y4 and MSC subjected to cytotoxic stress of dexamethasone, hypoxia, or both

**Figure 1:**
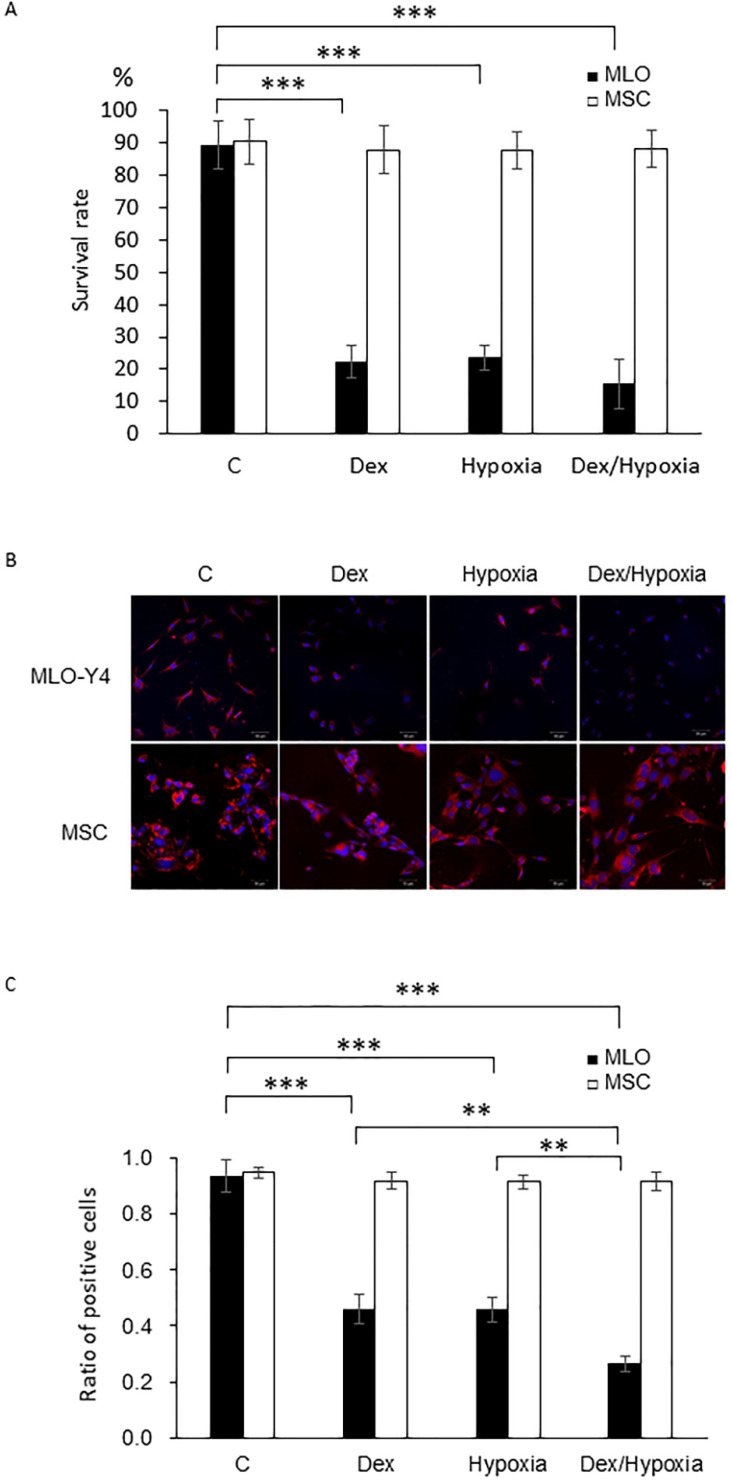
** Survival rates and mitochondrial membrane potentials of MLO-Y4 and MSC with the exposure to cytotoxic stresses. A.** Survival rates of MLO-Y4 and MSC after the exposure to dexamethasone (Dex group), hypoxia (Hypoxia group), or both dexamethasone and hypoxia (Dex/Hypoxia group). C group shows control groups without any exposure. MLO-Y4 shows decreased survival rates with the exposures; MSC shows resistance to them. **B.** Representative mitochondrial-membrane-potential-positive cells of MLO-Y4 and MSC under the treatment of Dex, hypoxia, or both. **C.** Ratios of number of mitochondrial-membrane-potential-positive cells divided by cell number stained with DAPI in MLO-Y4 and MSC under the treatment of Dex, hypoxia, or both. MLO-Y4 shows decreased mitochondrial membrane potentials with exposure to either Dex and hypoxia, and Dex plus hypoxia further decreased them; MSC maintains membrane potentials even with exposure to them. Black pillars show MLO-Y4 and white ones show MSC. **; means *p* < 0.01 and ***; *p* < 0.001.

MLO-Y4 showed decreased survival rates with exposure to either dexamethasone or hypoxia compared with those of the control group (82.0 ± 3.0 vs 23.0 ± 4.0, 20.0 ± 2.0, each ***; *p* < 0.001). Treatment with both dexamethasone and hypoxia further decreased survival of MLO-Y4, although this difference was not significant. In MSC, survival rates were maintained after the treatment with dexamethasone, hypoxia, or both (85.0 ± 4.0 vs 81.0 ± 1.0, 82.0 ± 2.0, 84.0 ± 6.0, each *p* > 0.05) (Fig. 1A).

### Mitochondrial membrane potentials of MLO-Y4 and MSCs under cytotoxic stresses of dexamethasone, hypoxia or both

In MLO-Y4, mitochondrial membrane potentials decreased, compared with those of the control, with either dexamethasone or hypoxia (93.5 ± 5.61 vs 45.82 ± 5.23, 45.92 ± 4.39, 26.58 ± 2.78 each ***; *p* < 0.001). Such a decreasing effect was significantly enhanced by treatment with both dexamethasone and hypoxia (26.59 ± 2.78, **; *p* < 0.01). In MSC, in contrast, mitochondrial membrane potentials did not decrease with dexamethasone, hypoxia, or both (94.75 ± 1.89 vs 91.90 ± 2.87, 91.50 ± 2.39, 91.55 ± 3.31 each *p* > 0.05) (Fig. 1B, C).

### Decreased expression of ATP5A, an energy producing enzyme, under the cytotoxic stresses

**Figure 2:**
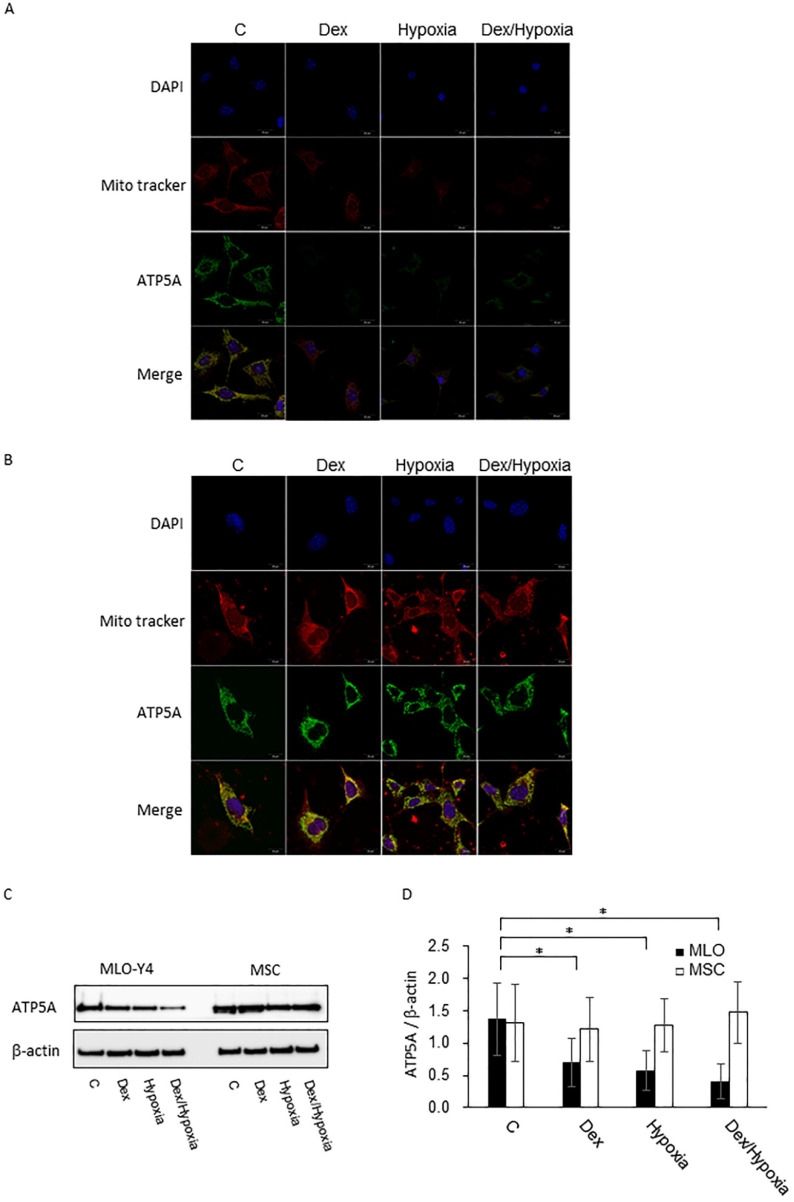
** Expression of ATP5A in MLO-Y4 and MSC with exposure to cytotoxic stresses. A and B** are representative immunofluorescent findings of DAPI, Mito tracker, ATP5A, and their merges with exposure to dexamethasone, hypoxia, or both dexamethasone and hypoxia as well as control in MLO-Y4 (A) and MSC (B), **C** shows representative findings of expression of ATP5A as well as β-actin of MLO-Y4 and MSC by western blot; **D** shows expression of ATP5A standardized by the expression of β-actin in MLO-Y4 (black pillars) and MSC (white pillars) with the exposure to dexamethasone, hypoxia, or both dexamethasone and hypoxia. *; means *p* < 0.05.

An ATP synthase, ATP5A, was demonstrated in the control groups of both MLO-Y4 and MSC, showing merged signals with Mito tracker red indicating its localization at the mitochondria. In MLO-Y4, expression of ATP5A decreased with exposure to dexamethasone, hypoxia and Dex plus hypoxia; In MSC, the expression as well as mitochondrial membrane potentials were maintained even when exposed to them (Fig. 2A and B). These effects in MLO-Y4 were demonstrated to be significant quantitatively by western blot (Fig. 2C and D).

### Induction of apoptosis by cytotoxic stresses through free-radical production, namely oxidative stress

**Figure 3:**
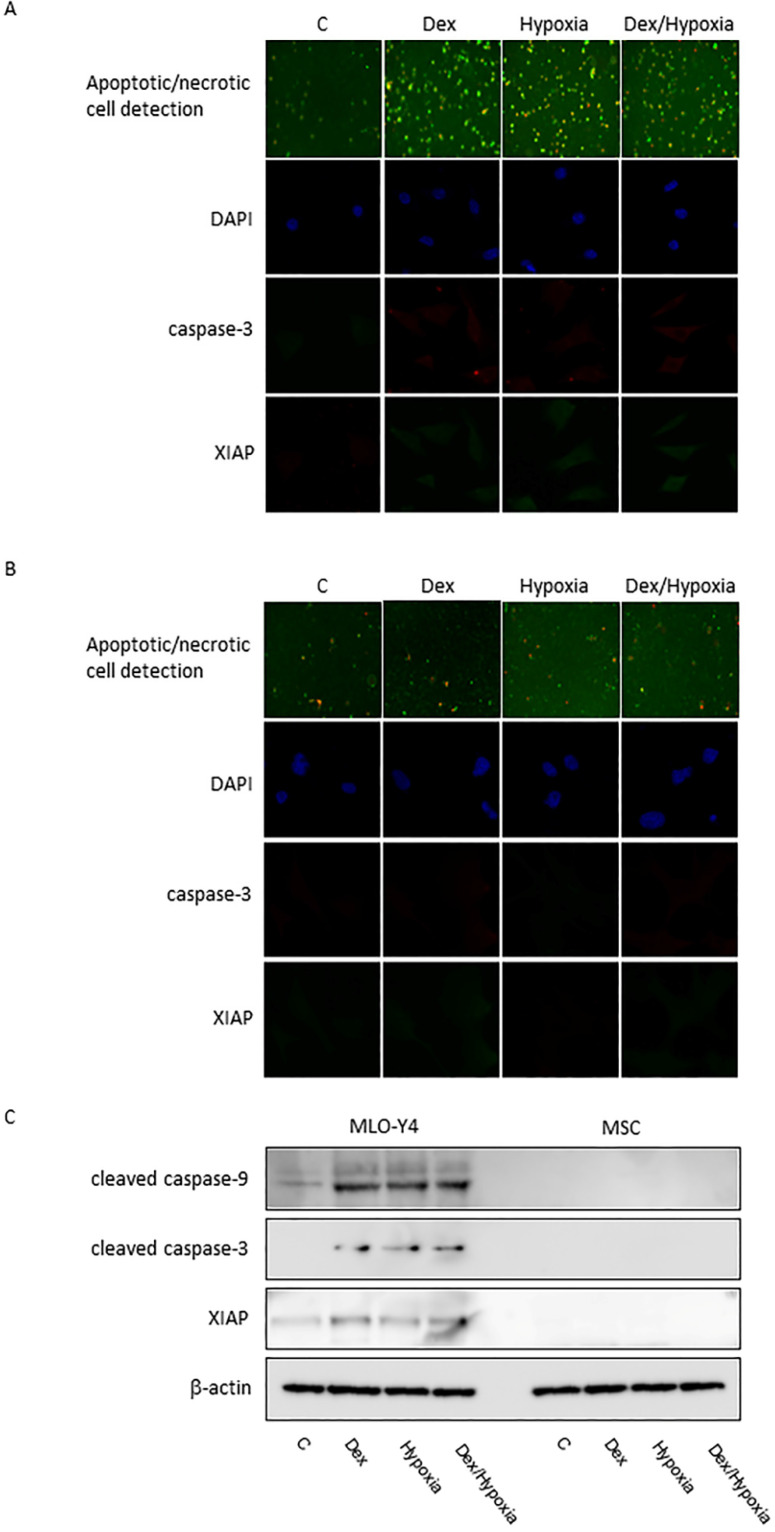
** Induction of apoptosis in MLO-Y4 and MSC. A and B** show immunofluorescent expression of apoptosis/necrosis, cleaved caspase-3, XIAP, as well as DAPI under the cytotoxic stresses dexamethasone, hypoxia, both, as well as control, in MLO-Y4 (A) and MSC (B). **C** shows western blots of cleaved forms of caspase-9 and -3 and X-linked inhibitor of apoptosis protein (XIAP) as well as β-actin in MLO-Y4 and MSC. MLO-Y4 shows activation of caspases and induction of apoptosis; apoptosis doesn't occur in MSC. Apoptosis was induced by cytotoxic stresses in osteocyte but not in mesenchymal stem cell.

**Figure 4:**
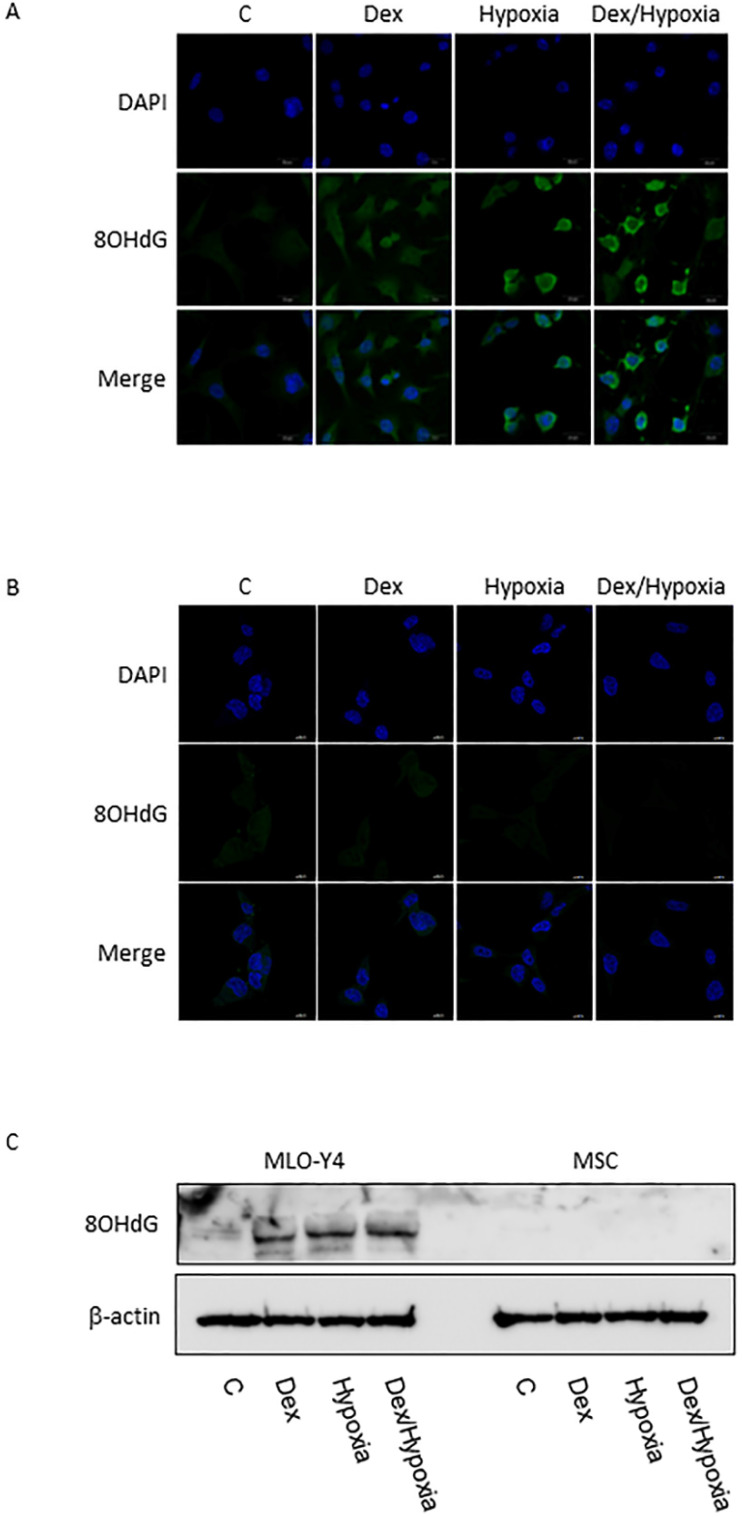
** 8-OHdG expression in MLO-Y4 and MSC. A and B** show immunofluorescent expression of 8-OHdG (8OHdG) as well as DAPI under the cytotoxic stresses dexamethasone, hypoxia, both, as well as control, in MLO-Y4 (A) and MSC (B). **C** shows western blots of 8-OHdG as well as β-actin in MLO-Y4 and MSC. MLO-Y4 shows activation of 8-OHdG; it was not detected in MSC even after the exposure to cytotoxic stresses. A predominant form of free-radical products was produced by the cytotoxic stresses in osteocyte but not in mesenchymal stem cell.

In MLO-Y4, induction of apoptosis by cytotoxic stresses was demonstrated with an Apoptotic/Necrotic Cells Detection Kit (Takara, Shiga, Japan) (Fig. 3A, B). Activation of caspase -3 was induced by cytotoxic stresses by both immunofluorescence and western blotting (Fig. 3A-C). A slight increase in the expression of XIAP, an inhibitor of apoptosis, was also seen with the exposures, but was not significant statistically (Fig. 3A-C). In MSC, no activation of caspase-9 and -3, or XIAP was demonstrated at all with the cytotoxic stresses, nor was induction of apoptosis detected (Fig. 3C). 8-OHdG, a predominant free-radical product, was produced by the cytotoxic stresses in MLO-Y4; it was not detected in MSC even after the exposure to cytotoxic stresses (Fig. 4A-C).

## Discussion

In glucocorticoid-induced osteonecrosis, remodeling of necrotic foci does not occur readily, and so in many cases the condition becomes progressive and eventually requires artificial joint replacement. Accordingly, the repair and regeneration of osteonecrotic tissues have become major topics of interest. Recently, the ability of MSC to mitigate the DNA injury associated with cell death (particularly apoptosis) and vascular injury, etc., has been highlighted 1, 13, 14. The implantation of MSC is also showing promise in the treatment of glucocorticoid-induced osteonecrosis because of its tissue repair and bone regenerative effects. However, much more still needs to be learned about the functional preservation of MSC in environments subjected to glucocorticoid-induced stress. This research was conducted with MSC and osteocyte cells placed in a hypoxic environment with added glucocorticoid. This model is said to relatively well reproduce the intraosseous events occurring at the time of glucocorticoid administration *in vivo*. By determining differences in stress resistance between the two, the possible role of MSC therapy in environments sufficiently stressful to cause osteocytic cell death was explored.

In osteocyte cells placed in a hypoxic environment to which glucocorticoid was added the preservation and function of mitochondria were lost, with an increase in oxidative stress and a state predisposing to cell death induced. Also, apoptosis of osteocyte cells was induced as shown by an increase in caspase. The mild elevation of XIAP observed was interpreted as a reactive increase to the protection of cell death. Accordingly, since clinically, the adjacent intraosseous area including the site sustaining osteonecrosis constitute a stressful environment, it was considered that the effects of interventions such as simple intraosseous bone implantation in the same sort of environment would be meager or even cause the development of new osteocytic cell death. In contrast, the mitochondria of the MSC placed in an environment exposed to cytotoxic stresses remained intact, and their function was preserved. The development of oxidative stress was thought to have been inhibited as well. The mitochondrial membrane potentials studied here were used as a marker of the course of mitochondrial apoptosis. Even when MSC were exposed to cytotoxic stresses their mitochondrial membrane potentials remained stable, with almost no apoptosis seen. Accordingly, even in the same intraosseous environment associated with osteocytic cell death *in vivo*, MSC seemed less likely to develop cell death such as mitochondrial apoptosis, in addition to which the possible preservation of energy production and other mitochondrial functions was also suggested. Furthermore, in the case of MSC, no changes in XIAP or caspase were evident. Since the mitochondria could be preserved even in an environment exposed to stress, this means that the impact of stress on them is minimal, namely, that they show sufficient resistance against the types of stresses to which they were exposed in the present experiment. In the normal mouse as well, it has been reported that *in vivo* bone marrow falls into a hypoxic state 15, whereas MSC in a similar hypoxic state continue to function normally. For this reason even when MSC are implanted intraosseously in a stressful environment it is not unreasonable to expect a full-fledged preservation of their function.

## Abbreviations

MSC: mesenchymal stem cell
QOL: quality of life
8-OHdG: 8-hydroxy-2'-deoxyguanosine
ATP: adenosine triphosphate
α-MEM: α-minimal essential medium
PBS: phosphate buffered saline
